# Linear elements are stable structures along the chromosome axis in fission yeast meiosis

**DOI:** 10.1007/s00412-021-00757-w

**Published:** 2021-04-07

**Authors:** Da-Qiao Ding, Atsushi Matsuda, Kasumi Okamasa, Yasushi Hiraoka

**Affiliations:** 1 Advanced ICT Research Institute Kobe, National Institute of Information and Communications Technology, 588-2 Iwaoka, Iwaoka-cho, Nishi-ku, Kobe, 651-2492 Japan; 2 Graduate School of Frontier Biosciences, Osaka University, Suita, 565-0871 Japan

**Keywords:** Meiosis, Linear elements, Chromosome, Fission yeast

## Abstract

The structure of chromosomes dramatically changes upon entering meiosis to ensure the successful progression of meiosis-specific events. During this process, a multilayer proteinaceous structure called a synaptonemal complex (SC) is formed in many eukaryotes. However, in the fission yeast *Schizosaccharomyces pombe*, linear elements (LinEs), which are structures related to axial elements of the SC, form on the meiotic cohesin-based chromosome axis. The structure of LinEs has been observed using silver-stained electron micrographs or in immunofluorescence-stained spread nuclei. However, the fine structure of LinEs and their dynamics in intact living cells remain to be elucidated. In this study, we performed live cell imaging with wide-field fluorescence microscopy as well as 3D structured illumination microscopy (3D-SIM) of the core components of LinEs (Rec10, Rec25, Rec27, Mug20) and a linE-binding protein Hop1. We found that LinEs form along the chromosome axis and elongate during meiotic prophase. 3D-SIM microscopy revealed that Rec10 localized to meiotic chromosomes in the absence of other LinE proteins, but shaped into LinEs only in the presence of all three other components, the Rec25, Rec27, and Mug20. Elongation of LinEs was impaired in double-strand break-defective *rec12*^−^ cells. The structure of LinEs persisted after treatment with 1,6-hexanediol and showed slow fluorescence recovery from photobleaching. These results indicate that LinEs are stable structures resembling axial elements of the SC.

**Supplementary Information:**

The online version contains supplementary material available at 10.1007/s00412-021-00757-w.

## Introduction

Sexual reproduction is accomplished through a special process of cell division called meiosis. Meiosis produces gametes through two rounds of consecutive chromosome segregation, which reduce the diploid set of homologous chromosomes to a haploid set in the gametes. One of the most important processes during meiosis is the recombination of homologous chromosomes inherited from each of the parent cells. Crossover recombination generates physical links between homologs and genetic variations in the offspring. Physical links called chiasmata are required for reductional segregation of homologous chromosomes during meiosis I (Baker et al. 1976; Carpenter 1994).

Upon entering meiosis, replicated chromosomes are organized in an axis-loop conformation by the meiotic cohesin complex. The axis structures along the chromosome are called axial elements (Page and Hawley 2004). Later, in many organisms, a synaptonemal complex (SC) nucleates in between the paired homologous chromosomes and eventually to their entire length. The image of SCs revealed by electron microscopy (EM) shows a tripartite structure composed of two lateral electron-dense bands (to which the axial elements are incorporated) and a transversely striated central region. The SC facilitates the transformation of crossovers into chiasmata and the establishment of crossover interference in some organisms (Zickler and Kleckner 2015). The components of axial elements are meiotic cohesins and non-cohesin components including SYCP2 and SYCP3 in mammalian cells, Red1 and Mek1 in budding yeast, HORMA-domain proteins, including Hop1 in yeast, ASY1 and ASY2 in Arabidopsis, and HIM-3 and HTP1-3 in *Caenorhabditis elegans* (reviewed in Page and Hawley 2004)*.*

Unlike many other organisms, the fission yeast *Schizosaccharomyces pombe* does not assemble canonical SC. Instead, it forms the so-called linear elements (LinEs), an electron-dense filamentous structure revealed by EM (Bahler et al. 1993; Molnar et al. 2003; Lorenz et al. 2004, 2006). LinEs are composed of four essential components: Rec10, which has limited homology with the axial element protein Red1 in *Saccharomyces cerevisiae*, and three small coiled-coil proteins—Rec25, Rec27, and Mug20. These three small proteins have no orthologs beyond *Schizosaccharomycetales* (Table 1) (Lorenz et al. 2004; Davis et al. 2008; Spirek et al. 2009; Estreicher et al. 2012; Fowler et al. 2013). All of the four LinE components are required for DNA double-strand break (DSB) formation and recombination (Lorenz et al. 2004; Davis et al. 2008; Estreicher et al. 2012; Fowler et al. 2013; Ma et al. 2017). In addition, there are several LinE-binding and/or regulation proteins. In contrast to the core components of LinEs, these proteins are well-conserved, similar to the axial element binding proteins in other organisms (Table 1) (Loidl 2006). The yeast Hop1 and Mek1 homologs exist in *S. pombe* and are localized on LinEs (Lorenz et al. 2004; Brown et al. 2018). No defects, or only subtle defects, have been observed in the formation of LinEs in Hop1 or Mek1 defective *S. pombe* cells (Lorenz et al. 2004; 2006). The conserved SUMO protein Pmt3 also localizes on LinEs transiently; the morphology of LinEs changes in the absence of SUMO ligase Pli1 (Spirek et al. 2009). Rec7 and Rec24 form foci on LinEs through interaction with Rec10 and are involved in Rec12-directed DSB formation (Lorenz et al. 2006; Spirek et al. 2009; Bonfils et al. 2011; Miyoshi et al. 2012).

**Table 1:** Summary of protein components of chromosome axis and LinEs in *S. pombe* during meiotic prophase

	Proteins	Size	Domains	Orthologs	
				*Saccharomyces cerevisiae*	*Homo sapiens*
Axis	Rec8	64.0 kDa, 561 aa		REC8	REC8
	Rec11	107.4 kDa, 923 aa		IRR1, SCC3	STAG1,2,3
	Psm3	136.9 kDa, 1194 aa		SMC3	SMC3
	Psm1	140.5 kDa, 1228 aa		SMC1	SMC1
Axis binding or regulating	Pds5	138.9 kDa, 1205 aa		PDS5	PDS5A, 5B
	Wpl1	67.6 kDa, 602 aa		RAD61	WAPL
LinEs	Rec10	89.9 kDa, 791 aa	Coiled-coil (760–782) ^a^	RED1^b^	
	Rec25	17.1 kDa, 150 aa	Coiled-coil (83–103)		
	Rec27	14.8 kDa, 134 aa	Coiled-coil (54–113)		
	Mug20	17. 2 kDa, 151 aa	Coiled-coil (73–93, 104–124)		
LinE binding or regulating	Hop1	61.6 kDa, 528 aa	HORMA (17–127)	HOP1	HORMAD1, 2
	Mek1	51.1 kDa, 445 aa		MEK1	STK33, CHEK2, PHKG1,2, MYLK3, STK17A,17B
	Pmt3	12.9 kDa, 117 aa		SMT3	SUMO1,2,3,4
	Pli1	80.7 kDa, 727 aa		SIZ1, NFI1	PIAS1,2,3,4
	Rec7	38.3 kDa, 339 aa		REC114	REC114
	Rec24	40.3 kDa, 350 aa		MEI4	MEI4

^a^Lorenz et al. (2004)

^b^Similarity limited to a 64-amino acid region (Lorenz et al. 2004)

Source and references: PomBase (https://www.pombase.org/)

Meiotic cohesins are essential for sister chromatid cohesion and the establishment of axial elements (Page and Hawley 2004). Similarly, the establishment of LinEs requires a meiotic cohesin axis. In *S. pombe* meiosis, a large part of the mitotic cohesin subunits Rad21 and Psc3 are replaced by two meiotic cohesin subunits, Rec8 and Rec11 (Table 1) (Parisi et al. 1999; Watanabe and Nurse 1999; Yokobayashi et al. 2003). Furthermore, two conserved cohesin-associated proteins, Pds5 and Wpl1, are involved in the maintenance of the sister chromatid cohesion (van Heemst et al. 1999; Hartman et al. 2000; Panizza et al. 2000; Tanaka et al. 2001; Bernard et al. 2008) (Table 1). In *S. pombe*, chromosomes become less compacted in the absence of Rec8 or Rec11, whereas the loss of Pds5 or Wpl1 results in Rec8-dependent over-compaction (Ding et al. 2006; Sakuno and Watanabe 2015; Ding et al. unpublished observation). Using three-dimensional super-resolution structured illumination microscopy (3D-SIM), we have shown that Rec8 forms a linear axis on chromosomes, and this is required for the organized axial structure of chromatin during meiotic prophase; in the absence of Pds5, the Rec8 axis is shortened while the chromosomes are widened (Ding et al. 2015). LinE formation on chromosomes requires meiotic cohesin Rec8 and Rec11 (Molnar et al. 1995, 2003). Rec11 phosphorylation by casein kinase 1 recruits Rec10 to the chromosome (Sakuno and Watanabe 2015; Phadnis et al. 2015).

The fine structure of LinEs has been examined by EM as well as immunofluorescence microscopy in spread cells. LinEs are observed as dots or short single lines at an early stage; then, they become longer lines or bundles at late meiotic prophase (Bahler et al. 1993; Molnar et al. 2003; Lorenz et al. 2004, 2006; Davis et al. 2008). Live cell imaging of LinE components tagged with GFP or other fluorescent proteins has shown dot-like foci or linear structures in intact cells (Fowler et al. 2013). Both EM and conventional live cell imaging remain problematic. First, the structure of LinEs may not be well preserved or may even be reorganized during nuclear spreading for EM specimen preparation. Second, the resolution of live cell fluorescence microscopy is not high enough to reveal the fine structure of LinEs. In this study, we examined LinEs using 3D-SIM with the aim of elucidating its fine structure and dynamics in live meiotic cells. Our results showed that LinEs are discontinuous threads formed along chromosome axes during meiotic prophase.

## Materials and methods

### Strains and culture

The *S. pombe* strains used in this study are listed in Table s1. *Schizosaccharomyces pombe* standard culture media YES, ME, and EMM2-N were used for routine culture, meiosis induction, and live observation, respectively. GFP or mCherry tagging and gene deletions were created using a PCR-based gene-targeting method (Bahler et al. 1998). For Rec10, Rec25, Rec27, and Mug20-GFP or mCherry tagging, the open reading frame of GFP or mCherry was integrated at the C-terminal end of the endogenous gene locus in the genome. The Hop1-GFP tagging and Hop1 deletion strains were purchased from Japan National BioResource Project (https://yeast.nig.ac.jp/yeast/top.xhtml); the *hop1-*GFP-fusion gene was inserted at the *lys3* locus in addition to the endogenous gene.

A CellASIC™ ONIX Microfluidic Perfusion System (EMD Millipore Corporation, Billerica, MA, USA) (Bisson-Filho et al. 2017) and Y04 microfluidic plates for yeast cells (CellASIC Onix) were used for 1,6-hexanediol treatment at the microscope stage. Cells were suspended in EMM2-N and loaded into the trapping region of a microfluidic plate. Thereafter, the EMM2-N flow rate was maintained at 12 μl/h and the chamber refresh time was about 1 min, according to the manufacturer’s instructions. Ten percent 1,6-hexanediol in EMM2-N was used for the treatment. The solution exchanging was manually controlled by clicking on the bars of the inlet valves in the CellASIC ONIX FG flow controller software during time-lapse imaging. The experimental conditions for the 1,6-hexanediol treatment were the same as previously described (Ding et al. 2019).

### Microscopy

A DeltaVision Elite microscope (Global Life Sciences Solutions Operations UK LTD) with an objective lens × 60 UPlanXApo NA 1.42 Oil (Olympus), set up in a temperature-controlled room at 26 °C, was used for deconvolution microscopy. Images from 15 focal planes at 0.3 μm intervals were taken every 5 min. Data analysis was carried out using the softWoRx software (Global Life Sciences Solutions Operations UK LTD) on a DeltaVision system. For all live cell observations, cell suspensions were placed in 35-mm glass-bottom culture dishes (MatTek Corp., Ashland, MA, USA) coated with 0.2% (w/v) soybean lectin (Sigma).

A DeltaVision|OMX microscope version 2 (Global Life Sciences Solutions Operations UK LTD) with an objective lens × 100 UPlanSApo NA1.40 Oil (Olympus, Tokyo, Japan), or OMX SR with an objective lens × 60 PlanApoN NA1.42 Oil (Olympus, Tokyo, Japan), was used for 3D-SIM imaging. For live cell SIM, cells in EMM2-N attached to glass-bottom dishes were imaged with immersion oil with a refractive index of 1.522 for OMX version 2 and 1.516 for OMX SR. Live cell SIM reconstruction was performed using the softWoRx software with a Wiener filter constant of 0.012 and triangle apodization. We obtained a series of optical transfer functions (OTFs) with varying amounts of spherical aberration and used the OTF that produced the best contrast after 3D-SIM reconstruction (Demmerle et al. 2017). A set of 9 to 17 optical sections was taken at 0.125-μm focus intervals. For simultaneous observations of Rec10-mCherry co-stained with Rec8-GFP, or Rec10-GFP co-stained with H2B-mCherry, each of the two through-focus sets of 9 optical sections was collected separately for each wavelength. Because mCherry is bleached by 488-nm illumination, mCherry was imaged first, and then GFP was subsequently imaged without delay (Fiolka et al. 2012). Our custom software *Chromagnon* was used for correction of chromatic aberrations and camera alignment (Matsuda et al. 2018; https://github.com/macronucleus/Chromagnon).

Photobleaching and recovery experiments were conducted using the Photokinetics function of a DeltaVision|OMX microscope SR with an objective lens × 60 PlanApoN NA1.42 Oil (Olympus, Tokyo, Japan). We used a 488-nm laser, 10% of maximum power, and duration of 0.01 s to bleach the GFP fluorescence signal. The FRAP data analysis was performed using the PK analysis function of the softWoRx software with the two-component model.

## Results

### LinE proteins cooperatively form a filamentous structure in living cells

**Fig. 1:**
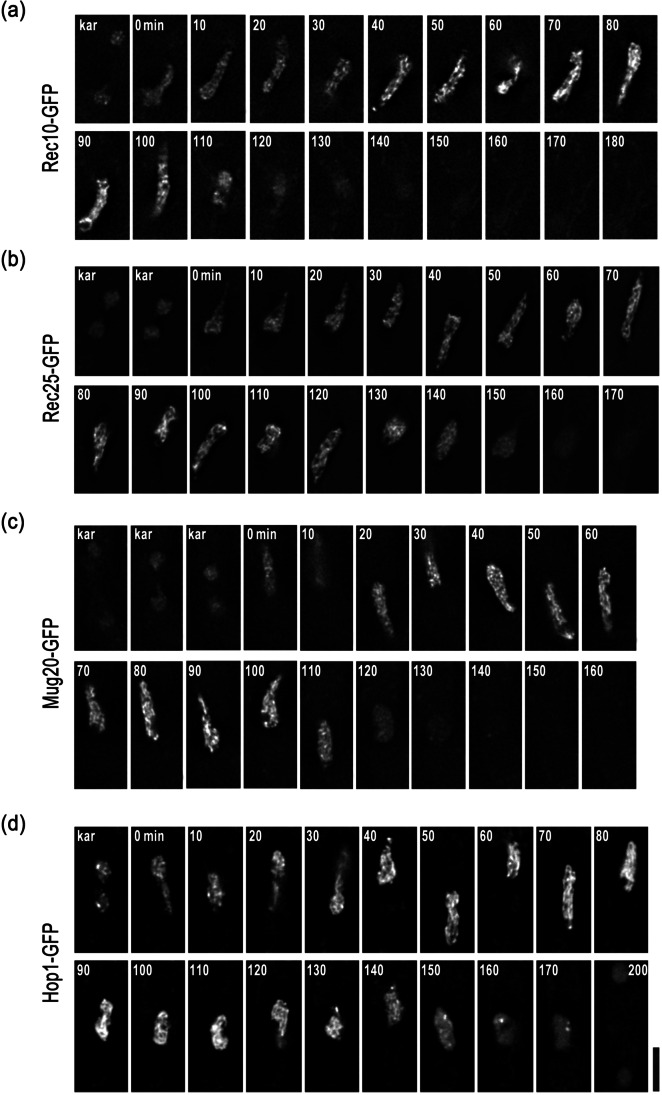
LinEs appear from karyogamy and disappear at the end of the horsetail stage. Selected time-lapse images of live cell observations of the LinE proteins in wild-type cells. **a** Rec10-GFP. **b** Rec25-GFP. **c** Mug20-GFP. **d** Hop1-GFP. Label “kar” stands for karyogamy. Numbers indicate the time in minutes after karyogamy. Each image is a single optical section from 3D deconvolved stacks collected at the indicated time points. Scale bar represents 5 μm, which applies to **a**-**d**

To follow the dynamics of LinEs during meiosis, we performed live cell imaging with GFP-tagged Rec10, Rec25, Rec27, Mug20, and Hop1. The results confirmed that GFP-fusion strains show fertilities and periods of meiotic prophase similar to those of the wild-type strain (Table 2). The results of live cell imaging are shown in Fig. 1 and Fig. s1a. Upon nitrogen starvation, haploid cells of the opposite mating type conjugate to form a zygote. Their nuclei fuse with each other (karyogamy, “kar” in Fig. 1) to form an elongated diploid nucleus called a “horsetail” nucleus (Fig. 1). The horsetail nucleus represents meiotic prophase in *S. pombe* (Chikashige et al. 1994; Ding et al. 2004). We found that all the core LinE proteins (Rec10, Rec25, Rec27, and Mug20) appeared during karyogamy and disappeared at the end of the horsetail stage (Fig. 1a-c; Fig. s1a). However, the GFP signal of the LinE-binding protein Hop1 did not disappear even after meiotic chromosome segregation I (Fig. 1d, 200 min), which contrasts with the core LinE proteins. All of the LinE proteins, as well as Hop1, displayed indistinguishable staining patterns in the horsetail nucleus where the GFP signals were not uniformly distributed but looked like numerous uneven dashed lines; the intensity of the GFP signals increased during the horsetail stage (Fig. 1; Fig. s1a).

**Table 2:** Spore formation and viability

	Normal 4 spore formation (number of zygotes examined)	Spore viability (number of spores examined)	Time of horsetail stage* (min, ± SD) (zygote number)
WT (L968)	98.0% (249)	94.3% (210)	
Rec10-GFP	94.0% (252)	90.0% (420)	131 ± 13 (21)
Rec25-GFP	84.4% (307)	92.6% (210)	136 ± 6 (15)
Rec27-GFP	90.6% (329)	89.8% (196)	130 ± 11 (17)
Mug20-GFP	83.7% (355)	93.8% (210)	126 ± 13 (16)
Hop1-GFP	86.8% (311)	90.5% (420)	137 ± 12 (11)
* ∆rec10*	35.0% (268)		
* ∆rec25*	20.0% (300)		
* ∆rec27*	54.7% (300)		
* ∆mug20*	27.4% (270)		
* ∆hop1*	65.1% (228)		

^*^The time length of horsetail is defined as the time after karyogamy to the stop of horsetail nuclear movement

**Fig. 2:**
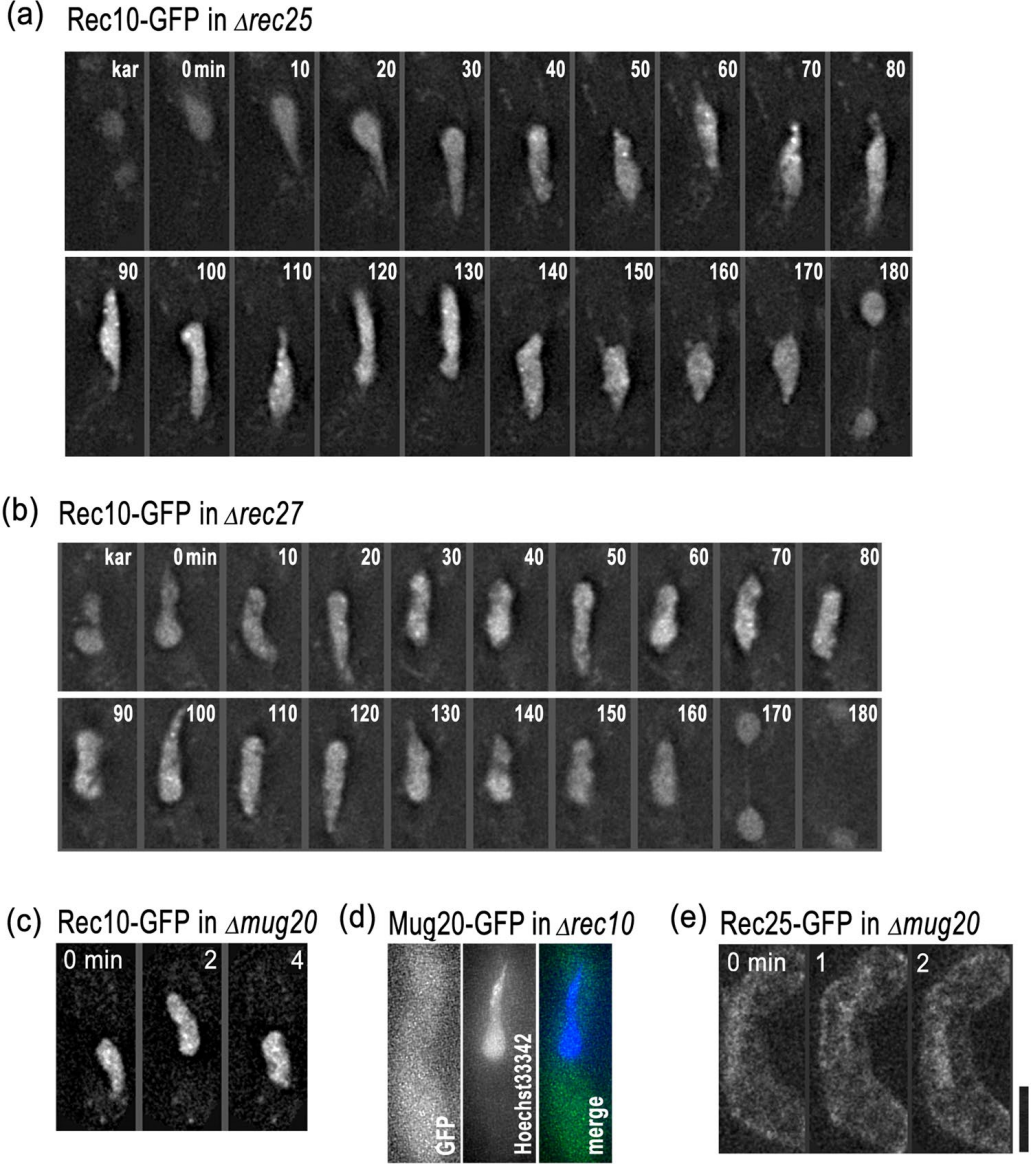
Formation of LinEs requires all the core components. Selected time-lapse images of live cell observations of the LinE proteins in various mutant backgrounds. **a** Rec10-GFP in a rec25-deletion cell. **b** Rec10-GFP in a rec27-deletion cell. **c** Rec10-GFP in a mug20-deletion cell. **d** Mug20-GFP in a rec10-deletion cell; the nucleus was stained with Hoechst33342. **e** Rec25-GFP in a mug20-deletion cell. Label “kar” stands for karyogamy. Each image is a single optical section from 3D deconvolved stacks collected at the indicated time points. Scale bar represents 5 μm, which applies to **a**-**e**

It has been shown that LinE components are mutually interdependent in their loading to chromosomes (Davis et al. 2008; Fowler et al. 2013). To address their interdependence more clearly, we followed the formation of LinEs in various deletion backgrounds. In the absence of Rec25, Rec27, or Mug20, Rec10-GFP fluorescence distributed to the entire nucleus without forming LinE-like linear fragments (Fig. 2a-c). Later, using 3D-SIM observations, we found that Rec10-GFP was actually binding with chromosomes in these mutant backgrounds (see below, Fig. 4a). This Rec10 localization was sustained until the first meiotic segregation (Fig. 2a, b; more than 190 ± 18 min; mean ± SD, *n* = 10), which was not observed in wild-type cells (Fig. 1; about 148 ± 8 min (mean ± SD, *n* = 11)). In the absence of the LinE-binding protein Hop1, Rec10-GFP staining was similar to that in wild-type cells (Fig. s1b). These results suggest that Rec10 can form LinEs only in the presence of all the other core LinE components (Rec25, Rec27, and Mug20). On the other hand, Mug20-GFP was uniformly distributed in the entire zygote in a Rec10-deletion background (Fig. 2d), which suggests that Rec10 is required for concentrating Mug20, and presumably other LinE components, in the nucleus by retaining them on chromosomes. In addition, in the absence of Rec25, the Mug20-GFP signal was barely observed in the nucleus (Fig. 2e), which is consistent with a previous report (Fowler et al. 2013).

### Observation of fine structure of LinE in living cells shows that LinEs are axial structures formed on each chromosome

**Fig. 3:**
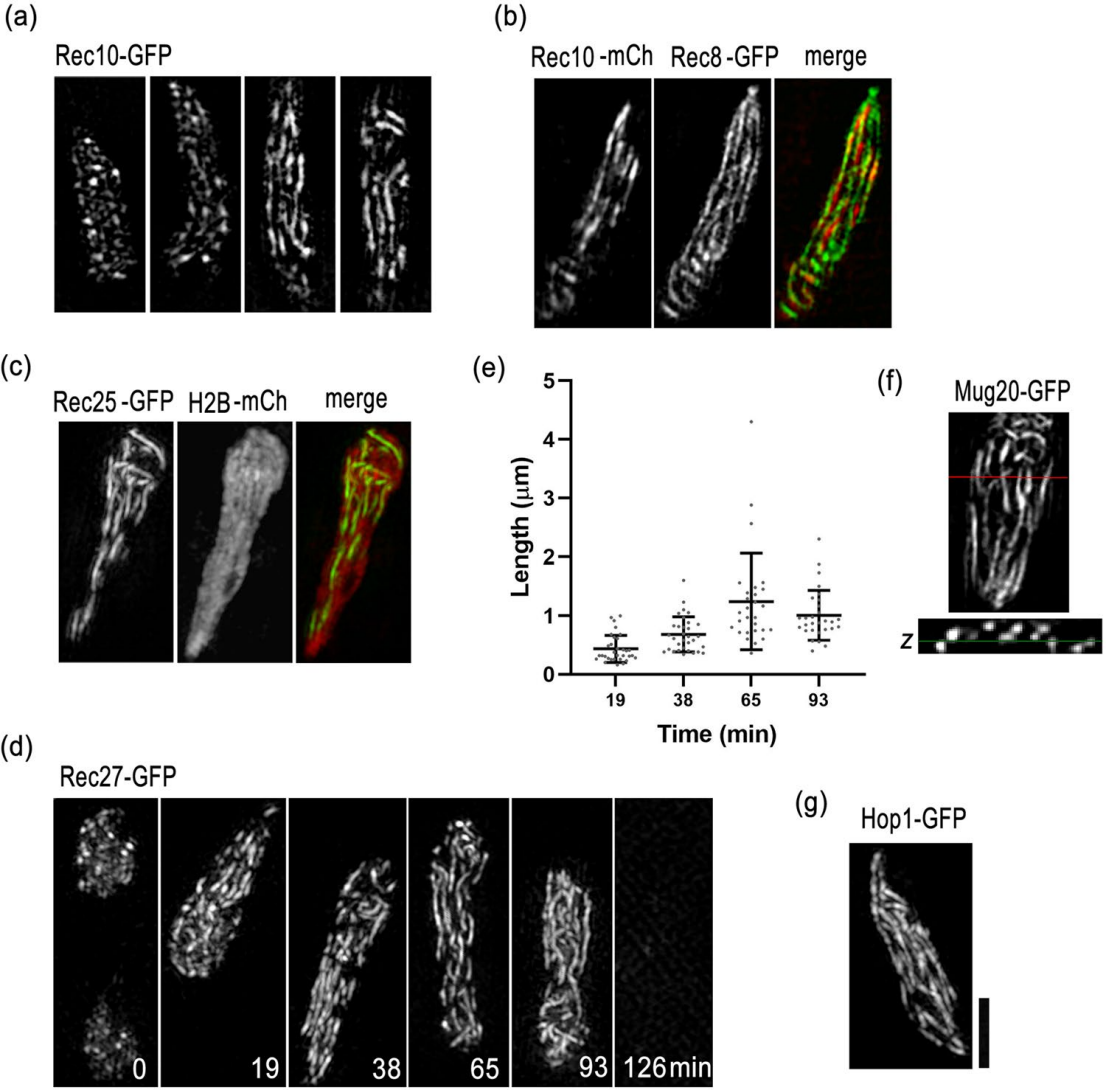
LinEs elongate during the horsetail stage. **a** Rec10-GFP in wild-type cells. Four representative single focus plane images from different cells in the horsetail stage are shown. **b** Double labeling of Rec10-mCherry and Rec8-GFP in a wild-type cell. Representative images from a single focus plane are shown. **c** Double labeling of Rec25-GFP and histone H2B-mCherry in a wild-type cell. Representative images from a single focus plane are shown. **d** Time-lapse observation of Rec27-GFP in a wild-type cell throughout the entire meiotic prophase. Each image is a projection from 3D stacks collected at the indicated time points. **e** Quantification of lengths of Rec27-GFP-labeled LinEs at the indicated time points as shown in **d**. Lengths of 28 to 32 LinEs in 3 horsetail nuclei measured in 3D are plotted. Error bars represent SD. **f** A representative XY plane image and its Z cross-sectional (the XZ plane) image of Mug20-GFP. The red line represents the cross-sectional position and the green line represents the XY plane. **g** A representative projected image of Hop1-GFP in a wild-type cell. All the images were obtained using 3D-SIM from living cells. Scale bar represents 2 μm, which applies to **a**-**d**, **f**, and **g**

To observe the structure of LinEs at higher resolution, we visualized GFP-tagged LinE components in live cells using the super-resolution 3D-SIM system. The fine structure of LinE observed by 3D-SIM in live cells is shown in Fig. 3. We found that LinEs in wild-type cells are short, discontinuous filamentous structures of various lengths (Fig. 3a). Double labeling of Rec10 with Rec8 (Fig. 3b), or Rec25 with histone (Fig. 3c), showed that LinEs were localized along the chromosomal axis in the horsetail nucleus. We also monitored the structural changes in LinEs using time-lapse 3D-SIM imaging. At karyogamy and the very beginning of the horsetail stage, LinE signals were scattered throughout the whole nucleus (Fig. 3d; Fig. s2). Then, after 15–20 min in the horsetail stage, a typical LinE structure appeared, and the LinEs elongated during progression in the horsetail stage (Fig. 3d; Fig. s2); the length of LinEs increased from 0.4 μm on average at 20 min to 1.2 μm on average at 65 min in the horsetail stage (Fig. 3e). At the end of the horsetail stage, the structure disappeared (Fig. 3d; Fig. s2). The number of LinEs counted from cross-sectional images (Fig. 3f) was about 8 to 12, indicating that LinEs are formed on each chromosome, but do not form in between homologous chromosomes (there are three pairs of looped homologous chromosomes in the horsetail nucleus; thus, the maximum number should be six at a cross section). These results are consistent with previous EM and immunostaining results, which have shown that short LinEs appear at the early stage and longer LinEs appear at the later stage, and the number of LinEs always exceeds the number of chromosomes (Bahler et al. 1993; Molnar et al. 2003; Lorenz et al. 2004, 2006; Spirek et al. 2009). Moreover, Hop1-GFP staining of LinEs was similar to other LinE components (Fig. 3g), as reported in previous studies using immunostaining (Lorenz et al. 2004, 2006; Brown et al. 2018).

### 3D-SIM analysis of LinEs in mutant cells

**Fig. 4:**
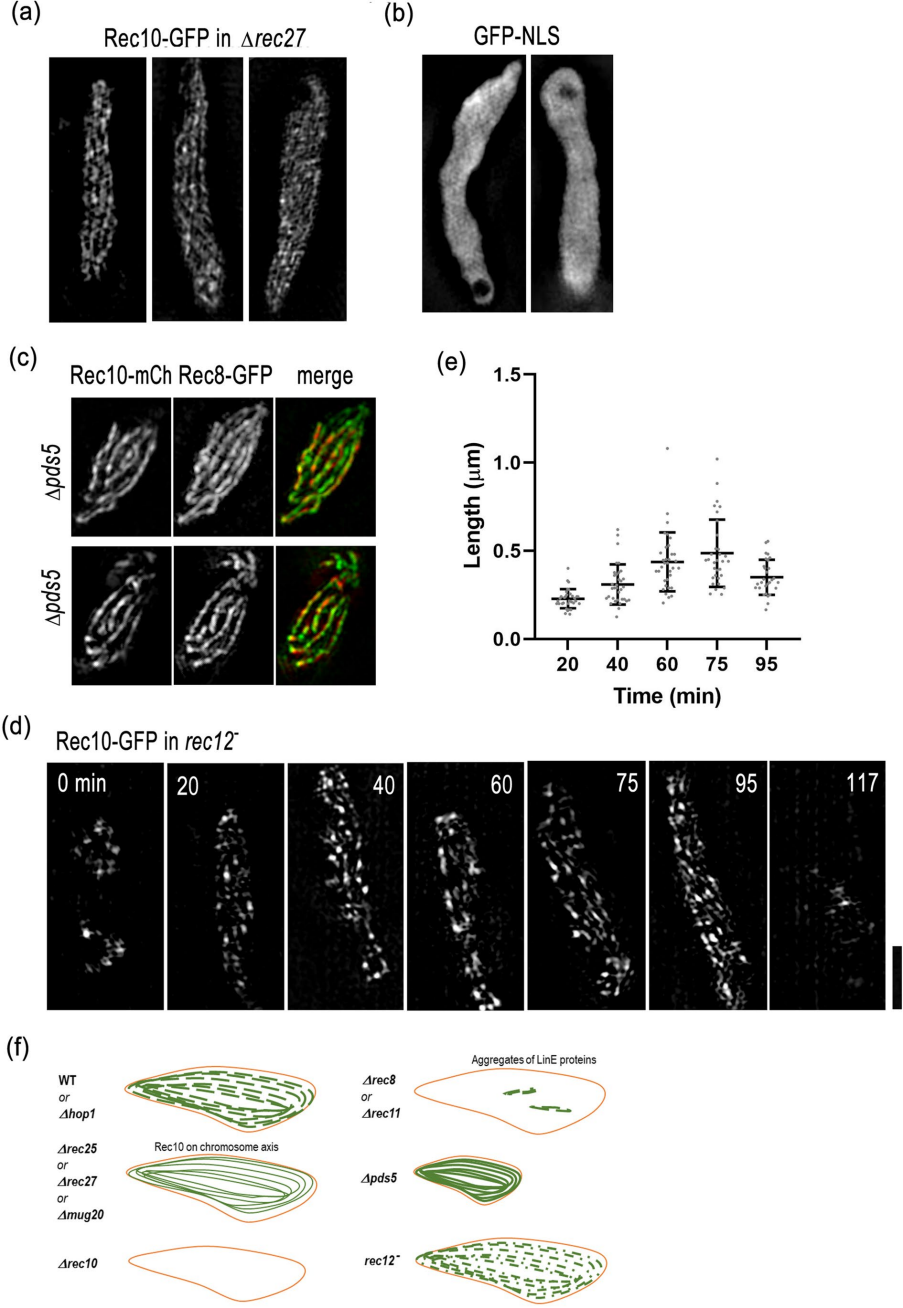
Aberrant localizations of Rec10 in mutant cells. **a** Representative single focus plane images of Rec10-GFP in three horsetail nuclei of rec27-deletion cells are shown. **b** Representative single focus plane images of GFP-NLS in two horsetail nuclei of wild-type cells are shown. **c** Representative single focus plane images from double labeling of Rec10-mCherry and Rec8-GFP in two horsetail nuclei of pds5-deletion cells are shown. **d** Time-lapse observation of Rec10-GFP in a *rec12*^−^ cell throughout the entire meiotic prophase. Each image is a projection from 3D stacks collected at the indicated time point. **e** Quantification of lengths of Rec10-GFP-labeled LinEs in *rec12*^−^ cells at the indicated time points as shown in **d**. Lengths of 29 to 34 LinEs at each time point in 3 horsetail nuclei measured in 3D are plotted. Error bars represent SD. **f** Graphic summary of LinE morphology in various mutant backgrounds. The green lines represent LinE while the orange line represents the nuclear envelope. The data showing LinE proteins form a few aggregates in the absence of Rec8 or Rec11 were adopted from published literature (Lorenz et al. 2004; Davis et al. 2008; Spirek et al. 2009; Fowler et al. 2013; Sakuno and Watanabe 2015). All the images were obstained using 3D-SIM from live cells. Scale bar represents 2 μm, which applies to a-d

Since in the absence of Rec25 or Rec27, Rec10-GFP showed a different labeling pattern compared to that in wild-type cells (Fig. 2a, b), we further studied its fine structure using 3D-SIM. We found that in Rec27 defective mutants, Rec10-GFP showed an axial staining pattern along the entire length of the horsetail nucleus, thinner than the staining patterns of histone or Rec8 (Fig. 4a, compared with Fig. 3b, c). The Rec10 localization in the absence of Rec27 appeared to be chromatin-bound rather than just localized to the nucleoplasm (Fig. 4a), since a freely distributed nuclear protein, GFP-NLS, which does not bind to chromosomes, showed a uniform staining pattern clearly distinct from Rec10-GFP in0020 *∆rec27* (Fig. 4b). In the absence of Pds5, chromosomes are compacted along the longitudinal axis (Fig. 4c; Ding et al. 2006). The LinEs in the *∆pds5* mutant had a more continuous appearance along the Rec8-axis (Fig. 4c, Rec10-mCherry) compared to that in the wild-type cells (Fig. 3b), reflecting chromosomal axis localization of LinEs.

We also observed the structure of LinEs in a DSB defective mutant, *rec12*^−^. We found no obvious differences in LinEs in the *rec12*^−^ background compared with wild-type cells using conventional wide-field microscopy (Fig. s3a). However, time-lapse observations using 3D-SIM revealed that LinEs were relatively short in *rec12*^−^ cells (Fig. 4d; Fig. s3b). The length of most LinEs in this mutant was 0.5 μm on average at 75 min (Fig. 4e), which is about half the length in wild-type cells (Fig. 3d, e; Fig. s2). This result is consistent with EM results, where the morphology classes IIb and III, which contain long LinE bundles or LinEs, were found to be rare in *rec12*^−^ cells (Molnar et al. 2003). These results suggest that DSB formation is required for the normal development of LinEs. A graphic summary of LinE formation or morphology in various mutant backgrounds is shown in Fig. 4f.

### LinEs have a stable structure

**Fig. 5:**
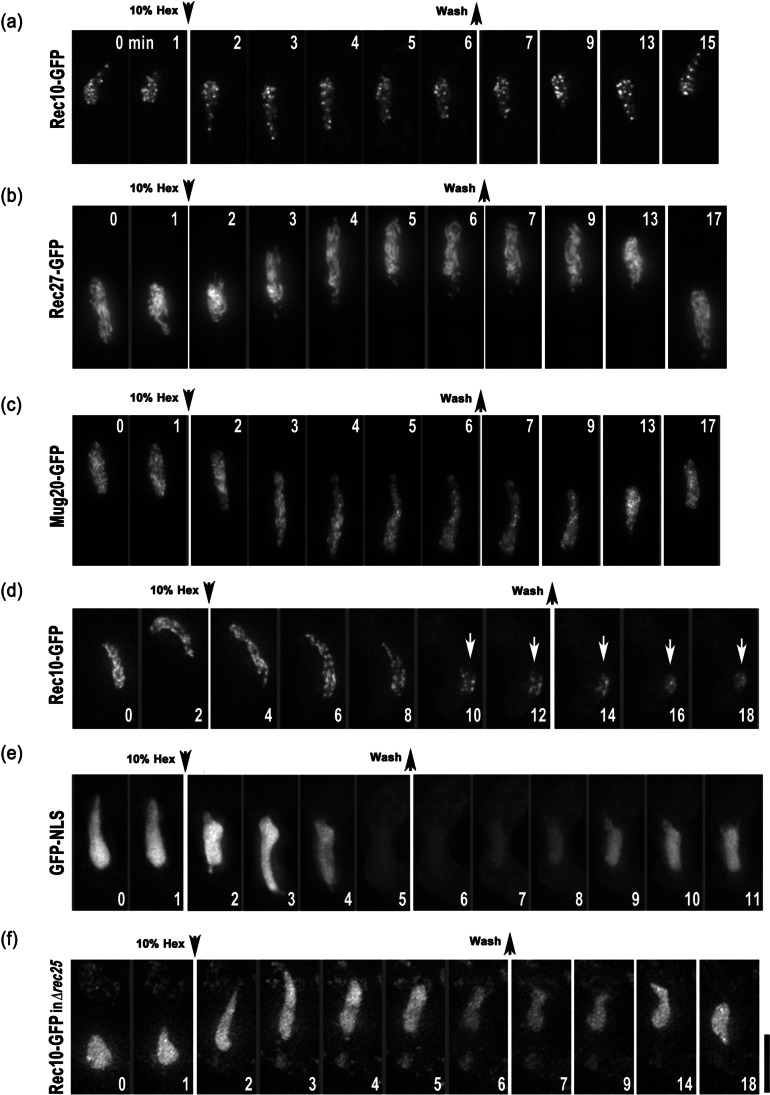
LinEs are stable under 1,6-hexanediol treatment. Time-lapse images of LinE proteins and GFP-NLS in **a**-**e** wild-type and **f** rec25-deletion living cells upon 10% 1,6-hexanediol treatment. 1,6-hexanediol was added or removed, as indicated by the black arrows. **a**-**c**, **f** Five-min treatment. **d** Ten-min treatment. **e** Four-min treatment. The numbers indicate the time (minute) of observation. Projected images from 3D deconvolved stacks in the horsetail stage are shown. White arrows in **d** indicate the round-shaped nucleus. Scale bar represents 5 μm, which applies to **a**-**f**

Research has demonstrated that the subunits of the SC in *Caenorhabditis elegans* are sensitive to aliphatic alcohols, and thus, have a liquid–liquid phase separation property (Rog et al. 2017). We have shown that lncRNA-protein complexes in *S. pombe* also exhibit phase separation properties, since 1,6-hexanediol treatment reversibly disassembled these complexes and disrupted the pairing of associated loci while the Rec8 axis resisted this treatment (Ding et al. 2019). Therefore, we examined if LinEs in *S. pombe* also have liquid droplet properties by continuously observing LinE proteins in living cells using a perfusion chamber. During live observations, the cells were briefly (5 min) treated with 10% 1,6-hexanediol. This treatment was longer than the 3-min treatment that disassembled lncRNA-protein complexes in *S. pombe* (Ding et al. 2019). However, GFP fluorescence of all of the LinE proteins showed little reduction by the addition of 10% 1,6-hexanediol for 5 min (Fig. 5a-b). Ten-minute incubation of the cells in 1,6-hexanediol resulted in a partial reduction in GFP signals of all the LinE-GFP fusions (Fig. 5d and Fig. s4). After a 10-min incubation in 1,6-hexanediol, the elongated horsetail nuclei shrunk to a small round shape (the arrows in Fig. 5d, Fig. s4); the nuclei did not recover to the normal horsetail shape even after 1 h in a culture medium without 1,6-hexanediol, suggesting that the physiology of the cells was disrupted by 1,6-hexanediol treatment for longer than 5 min. As a control, cells expressing GFP-NLS were treated with 10% 1,6-hexanediol for 4 min. 1,6-hexanediol is known to disrupt the permeability barrier of nuclear pore complexes by inhibiting the formation of phase-separated hydrogels in their FG domains (Shulga and Goldfarb 2003; Schmidt and Gorlich 2015). As expected, GFP-NLS was distributed throughout the entire cell body after treatment with 10% 1,6-hexanediol and relocated toward the nucleus quickly after removal of the drug (Fig. 5e). These results suggest that LinEs are not liquid–liquid phase-separated droplets which can be easily resolved in 1,6-hexanediol.

Furthermore, the stability of Rec10-GFP in the absence of other LinE components was examined. In the *∆rec25* background, Rec10-GFP was not sensitive to 1,6-hexanediol treatment (Fig. 5f), similar to in wild-type cells. Thus, Rec10-GFP seems to form a stable chromosome-bound structure even without other LinE components.

**Fig. 6:**
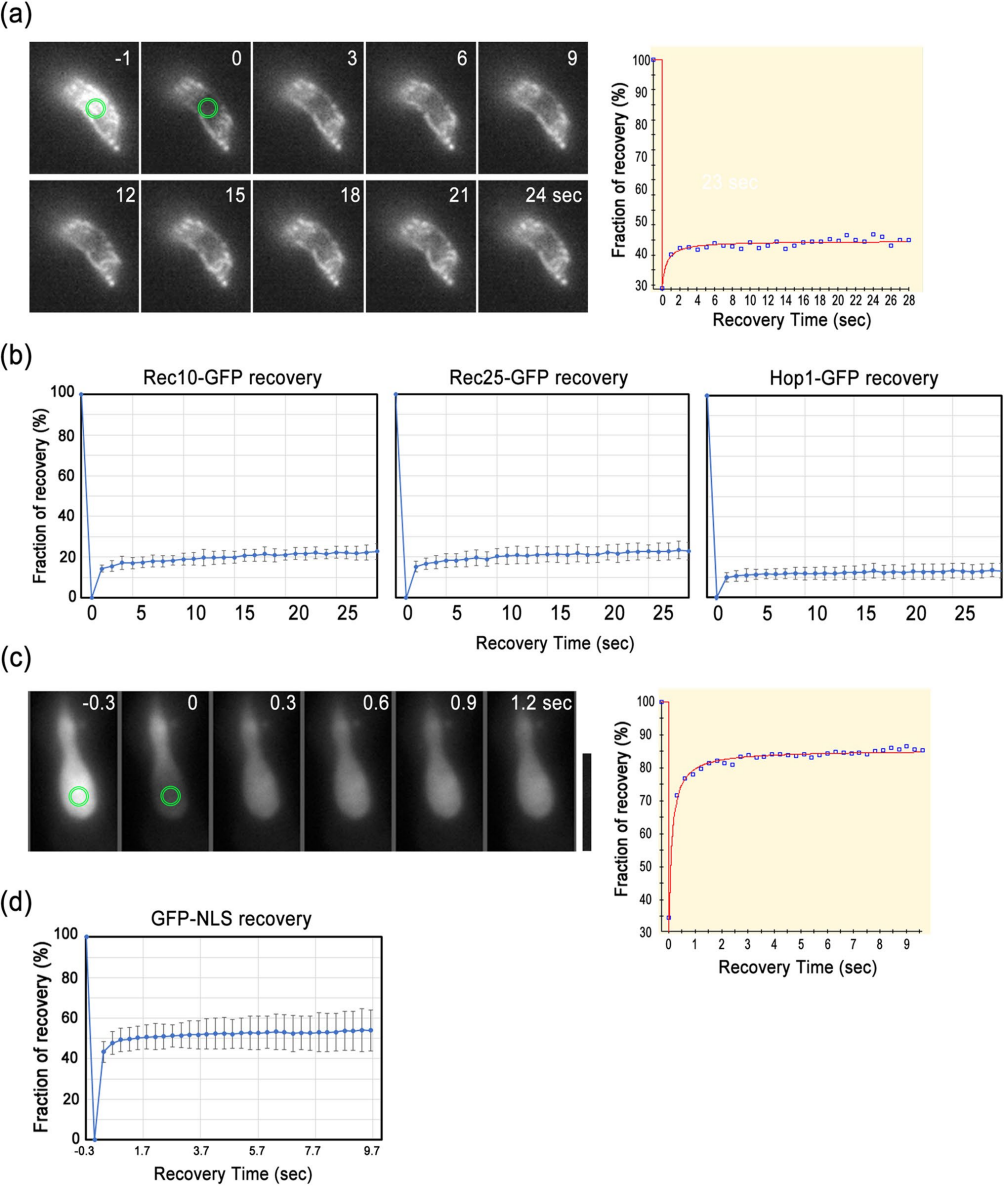
LinEs are stable structures as estimated by the FRAP experiment. **a** An example of a FRAP experiment showing Rec25-GFP before (− 1 s) and after photobleaching (0–23 s) at the target spot (green circles). The fluorescence recovery graph (right panel) plots the fluorescence intensity before and after the event. **b** Rec10-GFP, Rec25-GFP, and Hop1-GFP recovery after photobleaching. Each graph is the mean (± SD) from 10 cells. **c** An example of a FRAP experiment with GFP-NLS before (− 0.3 s) and after photobleaching (0–3 s) at the target spot (green circles). The fluorescence recovery graph (right panel) plots the fluorescence intensity before and after the event. **d** Mean (± SD) data from 10 cells for GFP-NLS recovery after photobleaching. Scale bar represents 5 μm, which applies to **a** and **c**

Another remarkable feature of liquid droplets is their rapid fluorescence recovery after photobleaching (FRAP). We then performed FRAP experiments and found that the fluorescence of GFP-tagged Rec10, Rec25, and Hop1 was not recovered 25 s after photobleaching (Fig. 6a, b). As a control, GFP-NLS recovered within 0.3 s after photobleaching (Fig. 6c, d). Taken together, these results suggest that LinEs are not liquid droplets or liquid crystals but relatively solid and stable proteinaceous complexes.

## Discussion

### Hierarchy of LinE establishment

In this study, we evaluated the dynamics and interdependence of LinE proteins in living cells during the entire meiosis process. Among the essential components of LinEs, i.e., Rec10, Rec25, Rec27, and Mug20, Rec10 was found to bind to chromosomes without other LinE components, whereas the other LinE components required Rec10 for their binding to chromosomes (Fig. 2d) (Fowler et al. 2013). It has been found that the C-terminus of Rec10 binds to CK1-phosphorylated meiotic cohesin Rec11 (Sakuno and Watanabe 2015; Phadnis et al. 2015). This phosphorylation-dependent recruitment of Rec10 to Rec11 may explain the chromosome-bound appearance of Rec10-GFP in the absence of other LinE components. Using super-resolution microscopy, side-by-side positionings of axial element proteins with cohesins have been precisely mapped in *C. elegans* (Kohler et al. 2017). The role of Rec10 in linking LinEs with meiotic cohesins in *S. pombe* might be analogous to the role of axial element proteins in *C. elegans*.

We showed that the typical filamentous structure formed only when all four essential LinE components were present. It has been shown that axial element proteins form coiled-coil homotetramer (Red1 in yeast) or heterotetramer (SYPC2:SYPC3 in mammals, ASY1:ASY2 in plant) filaments (West et al. 2019). Since all of the four LinE proteins have the coiled-coil motif (Table 1), potential inter-molecular interactions mediated by these motifs might be responsible for the formation of the stable LinE fibers in *S. pombe*.

All LinE components showed similar dynamics, such that they appeared in the nucleus from karyogamy and disappeared at the end of the horsetail stage, before meiosis I (Fig. 1a-c). Without the formation of LinEs, Rec10 persistently stayed in the nucleus even after meiosis I (Fig. 2a, b), which is similar to the dynamics of Hop1 (Fig. 1d) and meiotic cohesins (Ding et al. 2006; Sakuno and Watanabe 2015). There may be an active process to resolve the LinE complex to proceed to the next step of meiosis.

### Structure and dynamics of LinEs in living cells

Using 3D-SIM, we obtained images of LinEs in living cells with higher resolution at 120 nm in the lateral direction and 350 nm in the axial direction. Our results confirmed that LinEs in *S. pombe* exhibit an axial structure that is colocalized with the cohesin axis of chromosome (Bahler et al. 1993; Lorenz et al. 2004, 2006). Using 3D-SIM, we could not observe the so-called networks which have been clearly shown with EM analyses (Bahler et al. 1993; Molnar et al. 2003). However, using time-lapse 3D-SIM imaging, we confirmed that LinEs grow from dots or short fragments to long filaments up to 1.5 μm in wild-type cells, and that this elongation of LinEs largely depends on Rec12. These long LinEs may coincide with the thick bundles observed in EM or immunostaining (Bahler et al. 1993; Molnar et al. 2003; Lorenz et al. 2004). In the EM analyses, the thick bundles of LinEs were constantly observed at the late interphase in wild-type cells but not in *rec12*^−^ cells (Molnar et al. 2003), thus raising the possibility that the thick bundles result from homologous recombination which entangles chromosomes while preparing the spread chromosome samples. Since LinEs are critical for the formation of meiotic recombination hotspots (Fowler et al. 2013), the LinE components are probably regulated together with other protein factors which function as recombination machinery.

### Stability of LinEs

Rec27 and the *C. elegans* SC protein SYP-2 share DNA sequence similarity at their coiled-coil domains (Fowler et al. 2013), suggesting that LinEs may have similar properties to the components of SC. The proteins of transverse filament of SC, but not the proteins of chromosome axis, in *C. elegans*, *S. cerevisiae*, and *Drosophila melanogaster* can be rapidly dissolved in the presence of 1,6-hexanediol, suggesting that the structure of SC forms through liquid–liquid phase separation (Rog et al. 2017). We tested if LinEs have a liquid–liquid phase separation property by treating them with 1,6-hexanediol and also performing FRAP experiments. However, we found that LinEs exhibit very solid and stable properties. This stability suggests that LinEs represent axial elements along the chromosome axis, but not the transverse filaments in between homologous chromosomes. No SC-like transverse filaments have ever been found in *S. pombe*. The only structures observed in between homologous chromosomes are lncRNA/RNA-transcription termination protein complexes, which are liquid–liquid phase separation droplets that play an essential role in the recognition and pairing of homologous chromosomes (Ding et al. 2019). The liquid crystalline-like property of SC is proposed to play a direct role in crossover interference (Rog et al. 2017). Since no crossover interference exists in *S. pombe*, LinEs on the chromosome axis may play roles in crossover control different from that of SC (Loidl 2006; Yamada et al. 2017). Further study of SC-less organisms will provide more insights into the recombination control specific to meiosis.

## Supplementary Information

Below is the link to the electronic supplementary material.

Supplementary file1 (DOCX 20 KB)

Supplementary file2 (PDF 4415 KB)
