# Supplementary material for: Linear elements are stable structures along the chromosome axis in fission yeast meiosis
Source: Chromosoma. 2021 Apr 7;130(2-3):149–62. doi: 10.1007/s00412-021-00757-w (PMC8426239; doi:10.1007/s00412-021-00757-w)
Supplement: Supplementary file 1 — Supplementary file1 (DOCX 20 KB) [file 412_2021_757_MOESM1_ESM.docx]

**Supplemental table**

**Table s1. Strain list**

| Strain | Genotype | in Figure |
| --- | --- | --- |
| YY365 | *h^90^ ade6-216 leu1-32 lys1-131 ura4-D18 rec10^+^::GFP-kan^r^* | 1a, 3a, 5a, 5d, 6a, 6b, s2a |
| YW046 | *h^90^ ade6-216 leu1-32 lys1-131 ura4-D18 rec25^+^::GFP-kan^r^* | 1b,6b, s2b, s4 |
| YW047 | *h^90^ ade6-216 leu1-32 lys1-131 ura4-D18 rec27^+^::GFP-kan^r^* | 3d, 3e, 5b, s1a, |
| YY965 | *h^90^ ade6-216 leu1-32 lys1-131 ura4-D18 mug20^+^::GFP-kan^r^* | 1c, 3f, 5c, s4 |
| FY16786 | *h^+^ ade6-216 his2 leu1-32 lys3 ura4-D18 hop1^+^-GFP::lys3^+^* | 1d, 3g,6b |
| FY16787 | *h^-^ lys3 ura4-D18 hop1^+^-GFP::lys3^+^* | 1d, 3g,6b |
| YW603-1B | *h^-^ ade6-210 ∆hop1:: kan ^r^ rec10^+^::GFP-kan^r^* | s1b, s2c |
| YW603-2D | *h^+^ ∆hop1:: kan ^r^ rec10^+^::GFP-kan^r^* | s1b, s2c |
| YW044 | *h^90^ ade6-216 leu1-32 lys1-131 ura4-D18 rec10^+^::GFP-kan^r^ ∆rec25:: hyg^r^* | 2a, 5f |
| YW045 | *h^90^ ade6-216 leu1-32 lys1-131 ura4-D18 rec10^+^::GFP-kan^r^ ∆rec27:: hyg^r^* | 2b, 4a |
| YY940 | *h^90^ ade6-216 leu1-32 lys1-131 ura4-D18 rec10^+^::GFP-kan^r^ ∆mug20:: hyg^r^* | 2c |
| YY974-6A | *h^90^ ade6-216 leu1-32 lys1-131 ura4-D18 mug20^+^::GFP-kan^r^ ∆rec10:: ura4^+^* | 2d |
| YW048 | *h^90^ ade6-216 leu1-32 lys1-131 ura4-D18 rec25^+^::GFP-kan^r^ ∆mug20:: hyg^r^* | 2e |
| YY938-17 | *h^90^ ade6-216 leu1-32 rec8^+^::GFP-kan^r^ rec10^+^::mCherry:: hyg^r^* | 3b |
| YW273-5 | *h^90^ ade6-216 leu1-32 lys1-131 ura4-D18 rec25^+^::GFP-kan^r^ aur1^r^[::hta1^+^-htb1^+^-mCherry]* | 3c |
| GS643 | *h^90^ nmt1_pro_-GFP-NLS::lys1^+^* | 4b, 5e,6c, 6d |
| YY937-1 | *h^90^ ade6-216 leu1-32 rec8^+^::GFP-kan^r^ rec10^+^::mCherry:: hyg^r^ ∆pds5::LEU2* | 4c |
| YY362 | *h^90^ ade6-216 leu1-32 lys1-131 ura4-D18 rec10^+^::GFP-kan^r^ rec12-152::LEU2* | 4d, 4e, s3a, s3b |

**Supplemental figures**

**Fig. s1. LinEs appear from karyogamy and disappear at the end of the horsetail stage.**

Selected time-lapse images of live cell observations of Rec27-GFP in a wild-type cell (a) and Rec10-GFP in a hop1-deletion cell (b). Each image is a single optical section from 3D deconvolved stacks collected at the indicated time point. Scale bar represents 5 μm, which applies to (a) and (b).

**Fig. s2. Time-lapse 3D-SIM observations of linE proteins in living cells during meiotic prophase.**

Selected time-lapse images of Rec10-GFP and Rec25-GFP in wild-type (a, b) and Rec10-GFP in hop1-deletion zygotes (c) are shown. Each image is a projection from 3D stacks collected at the indicated time point. Scale bar represents 2 μm, which applies to (a)-(c).

**Fig. s3. Time-lapse observations of Rec10-GFP in *rec12^-^* living cells during meiotic prophase.**

Selected time-lapse images using a deconvolution microscope (a) and 3D-SIM (b) are shown. Each image is a single optical section from 3D stacks collected at the indicated time point. Scale bars represent 5 μm in (a) and 2 μm in (b).

**Fig. s4. LinEs are stable even under a 10 min 1,6-hexanediol treatment.**

Time-lapse images of Rec25-GFP and Mug20-GFP in wild-type living cells upon 10 min treatment with 10% 1,6-hexanediol treatment. 1,6-hexanediol was added or removed, as indicated by the black arrows. The numbers indicate the time (minute) of observation. Projected images from 3D deconvolved stacks in the horsetail stage are shown. White arrows indicate the round-shaped nucleus. Scale bar represent 5 μm.
