# Supplementary figures and images for: Linear elements are stable structures along the chromosome axis in fission yeast meiosis
Source: Chromosoma. 2021 Apr 7;130(2-3):149–62. doi: 10.1007/s00412-021-00757-w (PMC8426239; doi:10.1007/s00412-021-00757-w)

Figure s1

(a)

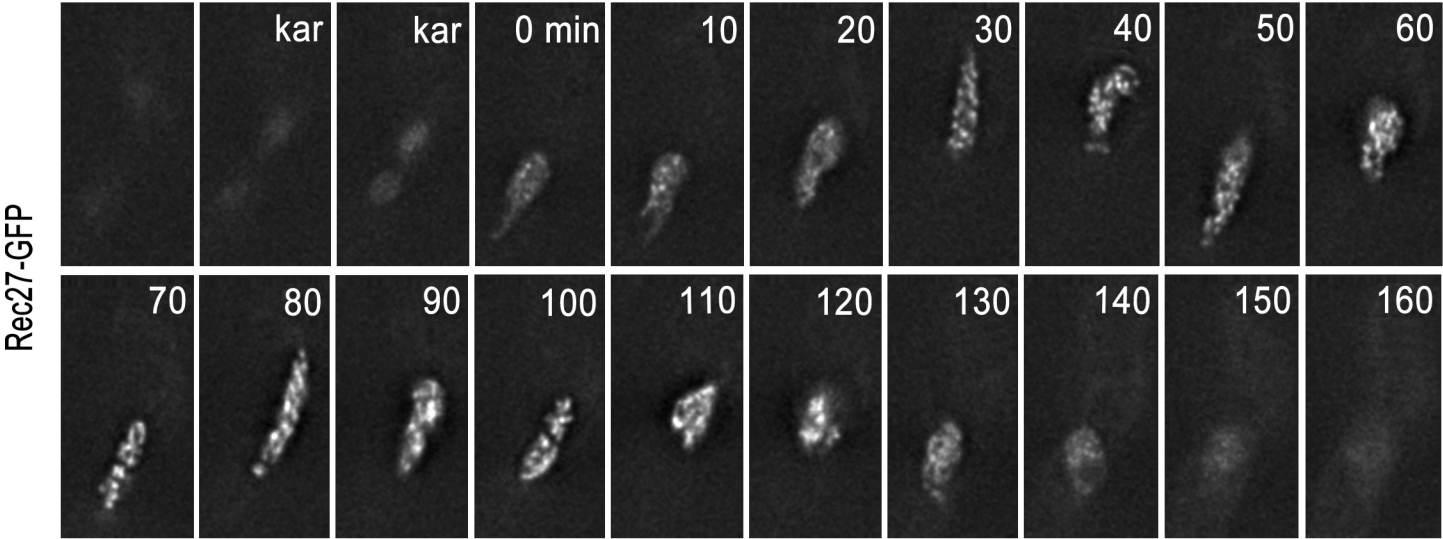

(b)

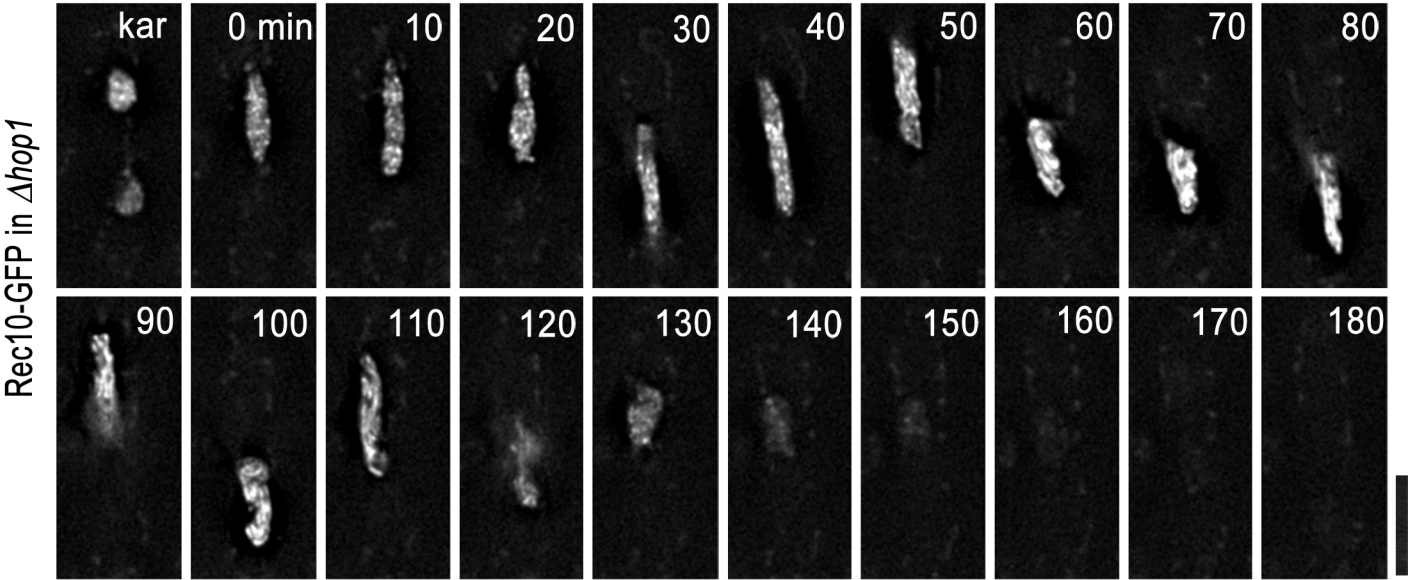

Figure s2

(a) Rec10-GFP

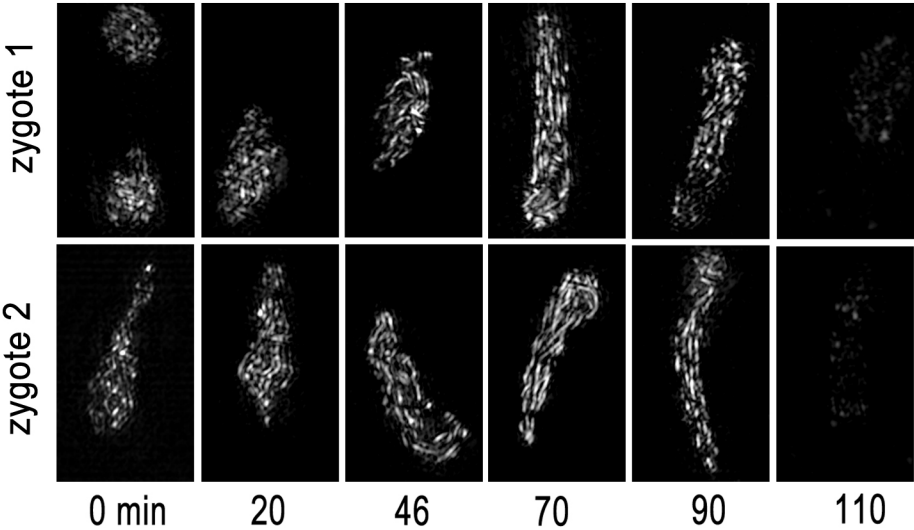

(b) Rec25-GFP

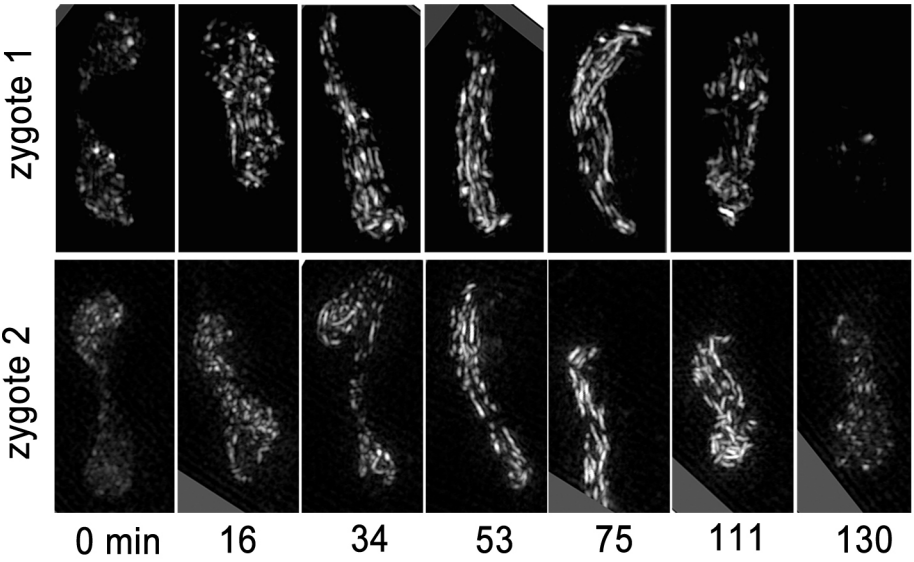

(c) Rec10-GFP in  $\Delta hop1$

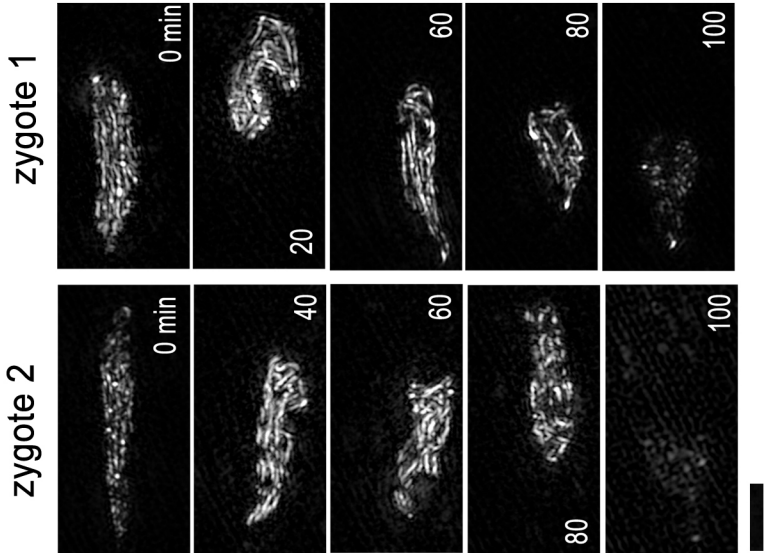

Figure s3

(a)

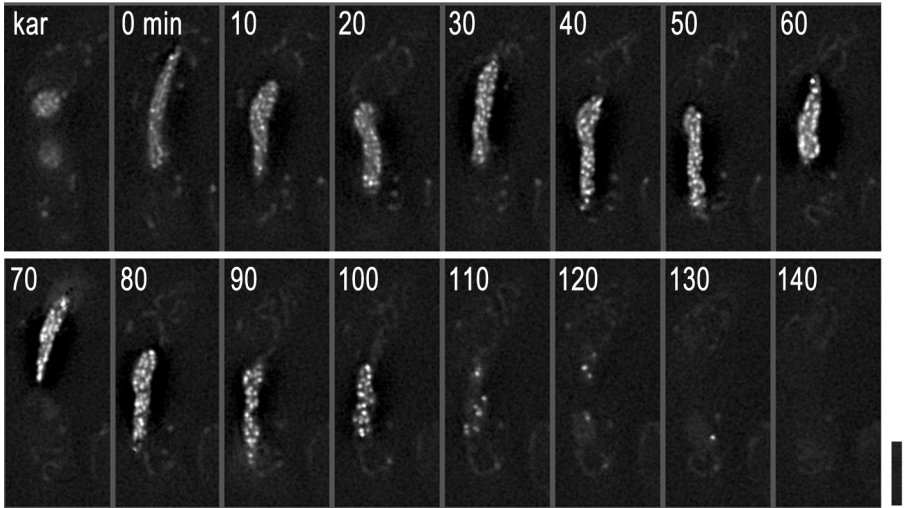

(b)

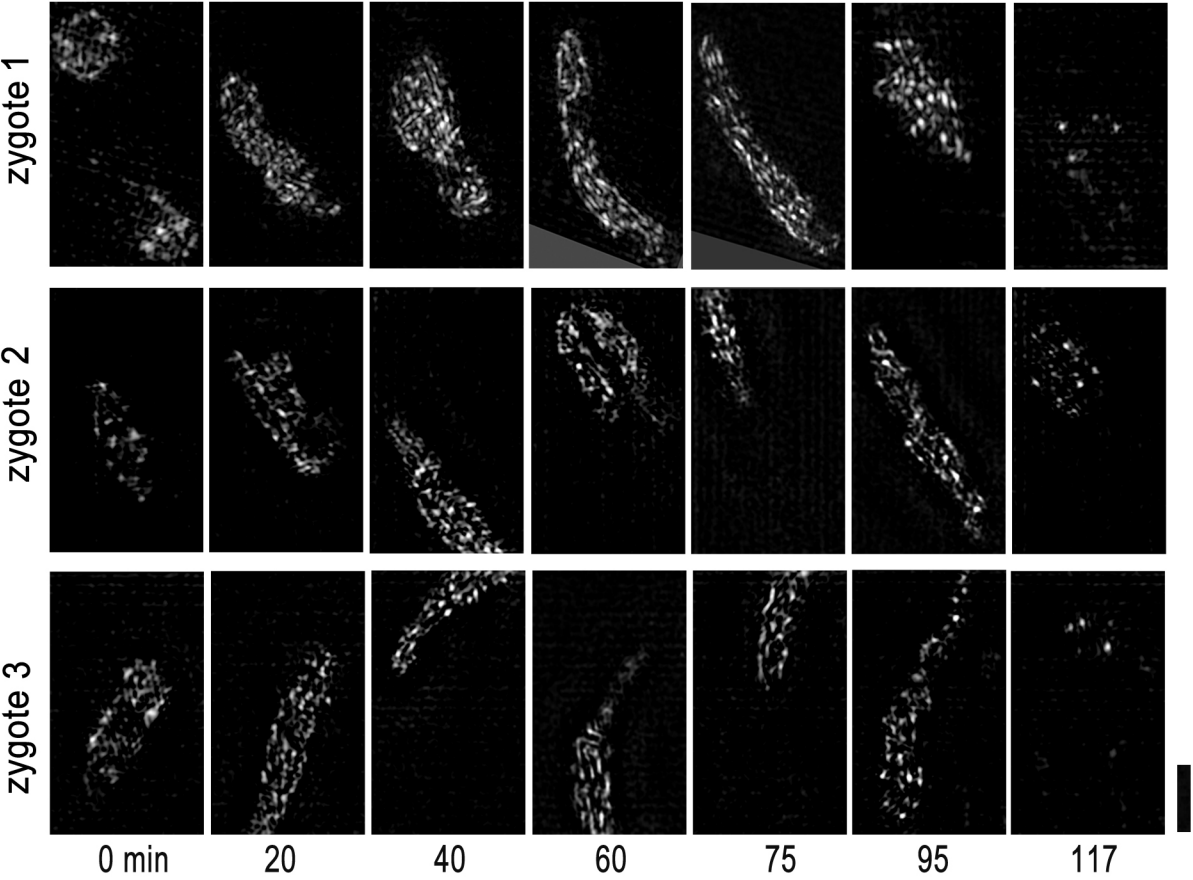

Figure s4

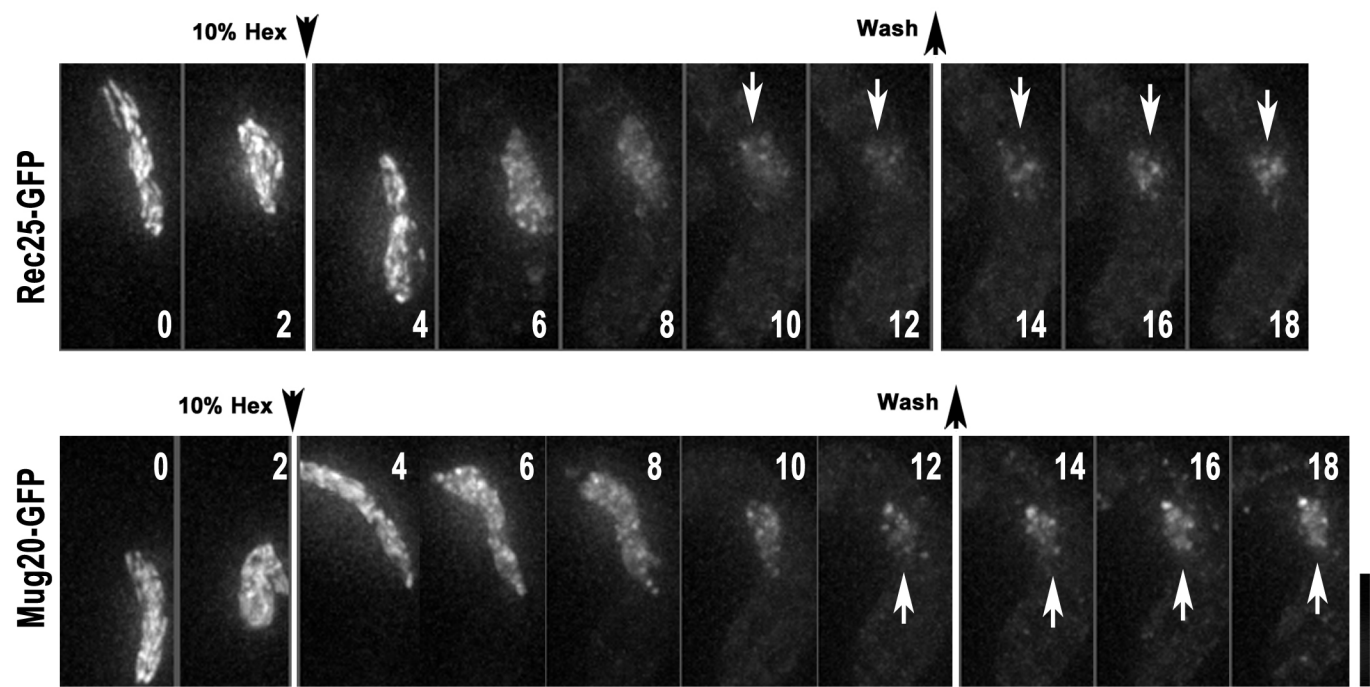

Supplement: Supplementary file 2 — Supplementary file2 (PDF 4415 KB) [file 412_2021_757_MOESM2_ESM.pdf]
